# The habitat connectivity hypothesis of escape in urban woodland birds

**DOI:** 10.1093/beheco/arac127

**Published:** 2023-02-10

**Authors:** Max Radvan, Anthony R Rendall, Michael A Weston

**Affiliations:** Deakin University Geelong, Australia, Centre for Integrative Ecology, School of Life and Environmental Sciences, Faculty of Science, Engineering and the Built Environment, Burwood Campus, VIC, Australia; Deakin University Geelong, Australia, Centre for Integrative Ecology, School of Life and Environmental Sciences, Faculty of Science, Engineering and the Built Environment, Burwood Campus, VIC, Australia; Deakin University Geelong, Australia, Centre for Integrative Ecology, School of Life and Environmental Sciences, Faculty of Science, Engineering and the Built Environment, Burwood Campus, VIC, Australia

**Keywords:** disturbance, economic escape theory, escape, habitat connectivity hypothesis, refuge

## Abstract

Habitat destruction and fragmentation increasingly bring humans into close proximity with wildlife, particularly in urban contexts. Animals respond to humans using nuanced anti-predator responses, especially escape, with responses influenced by behavioral and life history traits, the nature of the risk, and aspects of the surrounding environment. Although many studies examine associations between broad-scale habitat characteristics (i.e., habitat type) and escape response, few investigate the influence of fine-scale aspects of the local habitat within which escape occurs. We test the “habitat connectivity hypothesis,” suggesting that given the higher cost of escape within less connected habitats (due to the lack of protective cover), woodland birds should delay escape (tolerate more risk) than when in more connected habitat. We analyze flight-initiation distances (FIDs) of five species of woodland birds in urban Melbourne, south-eastern Australia. A negative effect of habitat connectivity (the proportion of the escape route with shrubs/trees/perchable infrastructure) on distance fled was evident for all study species, suggesting a higher cost of escape associated with lower connectivity. FID did not vary with connectivity at the location at which escape was initiated (four species), apart from a positive effect of habitat connectivity on FID for Noisy Miner *Manorina melanocephala*. We provide some support for two predictions of the “habitat connectivity hypothesis” in at least some taxa, and conclude it warrants further investigation across a broader range of taxa inhabiting contrasting landscapes. Increasing habitat connectivity within urban landscapes may reduce escape stress experienced by urban birds.

## Teaser

Many birds escape from humans as they do from predators, and it might be that birds in urban parks adjust their escape depending on the closeness of safe places such as trees. We show that birds flew further when disturbed where fewer trees occurred. This suggests that plantings in urban parks might influence how well people and birds coexist.

## INTRODUCTION

Urban expansion and associated habitat fragmentation increasingly bring humans into close proximity with wildlife including birds (Marzluff 2001). While urban environments typically limit avian diversity, some bird species persist in or exploit these highly altered landscapes (Chace and Walsh 2004). The majority of responses of birds evoked by close proximity with humans are escape responses, and humans are arguably treated by birds as if they were predators (Frid and Dill 2002). Therefore, part of successfully existing in urban environments inevitably involves adaptations which render interactions with humans sustainable, including adaptations of escape responses to humans (Samia et al. 2015).

During an “encounter” (i.e., when humans and wildlife come in close proximity; Weston 2019), the Economic Model of Escape posits that animals monitor the approaching stimulus (e.g., human) until it reaches a distance at which the animal perceives the cost of staying to be higher than the cost of fleeing; at that point the animal commences escape (Ydenberg and Dill 1986). Flight-initiation distance (FID; the distance at which an approaching stimulus evokes escape) is the most commonly used measure of the propensity to escape (Cooper and Blumstein 2015). Any factor which influences the costs and benefits of escape is therefore expected to alter FID (Ydenberg and Dill 1986). FID can be influenced by attributes of the stimulus, aspects of natural and life history of the responder, and prevailing environmental conditions (Schulte et al. 2004; Blumstein 2006; Weston et al. 2012; Hall et al. 2020). FID is also linked to post-initiation aspects of escape, such as the distance fled (Tätte et al. 2018). One key environmental attribute which has the potential to influence both FID and distance fled, and therefore may mediate propensity and intensity of escape, is the surrounding habitat in which an animal responds (Kopena et al. 2015; Hall et al. 2020).

Animals evolve escape tactics within specific habitats (Lima 1992), but because risk varies within the realized niche of most species, flexible escape responses are expected. For example, the local spatial distribution of habitat characteristics such as forest patches, open spaces and water bodies throughout the landscape may influence many aspects of escape deriving from spatial variation in detectability of predator and prey (Krams 2001; Boinski et al. 2003; Griesser and Nystrand 2009), escape route trajectory (Woodbury 1986; Zani et al. 2009) and availability of refuge (Cooper and Wilson 2007). While many studies have examined habitat as a mediator of escape response of birds, they do so in a comparative framework using coarse-scale categorical habitat factors, applied at the level of the species (e.g., wetlands, forests, grasslands; Blumstein 2006; Møller and Garamszegi 2012; Weston et al. 2021), or within species (e.g., urban, rural, and natural settings); the latter comparisons can confound human presence with habitat attributes (Valcarcel and Fernández-Juricic 2009; Lin et al. 2012; Weston et al. 2012). The nature of the local habitat in which an encounter occurs can influence escape responses in birds and reptiles (Schulte et al. 2004; Kopena et al. 2015; Hall et al. 2020; Maia-Carneiro et al. 2021). In the case of birds, no study known to us, apart from Fernández-Juricic et al. (2002) and Tätte et al. (2018), quantifies (as opposed to categorizes) habitat elements associated with escape initiation or escape trajectory. Studies of escape in reptiles often quantify contextual habitat elements such as distance to refuge.

### The habitat connectivity hypothesis of escape

Blumstein (2014) articulated the “Habitat Contiguity Hypothesis” of avian FID, whereby species in less contiguous habitats may be more likely to habituate (reduce FID) than those living in more contiguous habitats because those in more limited habitats have fewer options to move, and therefore must habituate. Here, we extend this novel yet untested hypothesis to a related, finer spatial scale, the scale at which individual escape decisions are made, and at which escape is conducted. Habitat connectivity influences how birds, especially woodland birds, move through landscapes at fine spatial scales (Taylor et al. 1993)—it presumably influences predator escape decisions and movements also. At the extreme end of the connectivity scale, some forest and woodland birds avoid crossing open habitat to move between habitat patches (Rodríguez et al. 2001; Robertson and Radford 2009). Given this, habitat connectivity may define escape options for many species of woodland birds. The “Habitat Connectivity Hypothesis of Escape” proposed here posits that habitat connectivity influences escape decisions in birds in several ways. Habitat connectivity is likely to alter the cost of escape because escape trajectories (i.e., routes) in less connected habitat likely involve longer movements to refuge (*sensu*Schulte et al. 2004). In turn, this alters the balance of costs and benefits involved in initiating escape, and when the cost to leave a patch increase, delays in departure are expected (Ydenberg and Dill 1986). Assessment of the costs of escape could conceivably be based on prevailing circumstances at the instant of escape, or based on a pre-planned or previously experienced escape trajectory (escape in birds appears to involve the cognitive capacity to plan escape in at least some species, as evidenced by some birds being more responsive the further they are from refuge; Guay et al. 2013; Dear et al. 2015).

We test predictions of the “habitat connectivity hypothesis of escape” in woodland birds, across a gradient of habitat connectivity. To avoid confounding between habituation and habitat, we confine our sampling to birds already exposed to high levels of human activity (i.e., urban woodland birds). Specifically, we predict that lower connectivity is associated with longer escape flights (i.e., distance fled) because suitable refuge is not available nearby. Consequently, because the costs of escape are comparatively higher where low connectivity exists, we predict that FIDs will be shorter in relatively poorly connected habitats.

## METHODS

### Study sites and species

**Figure 1:**
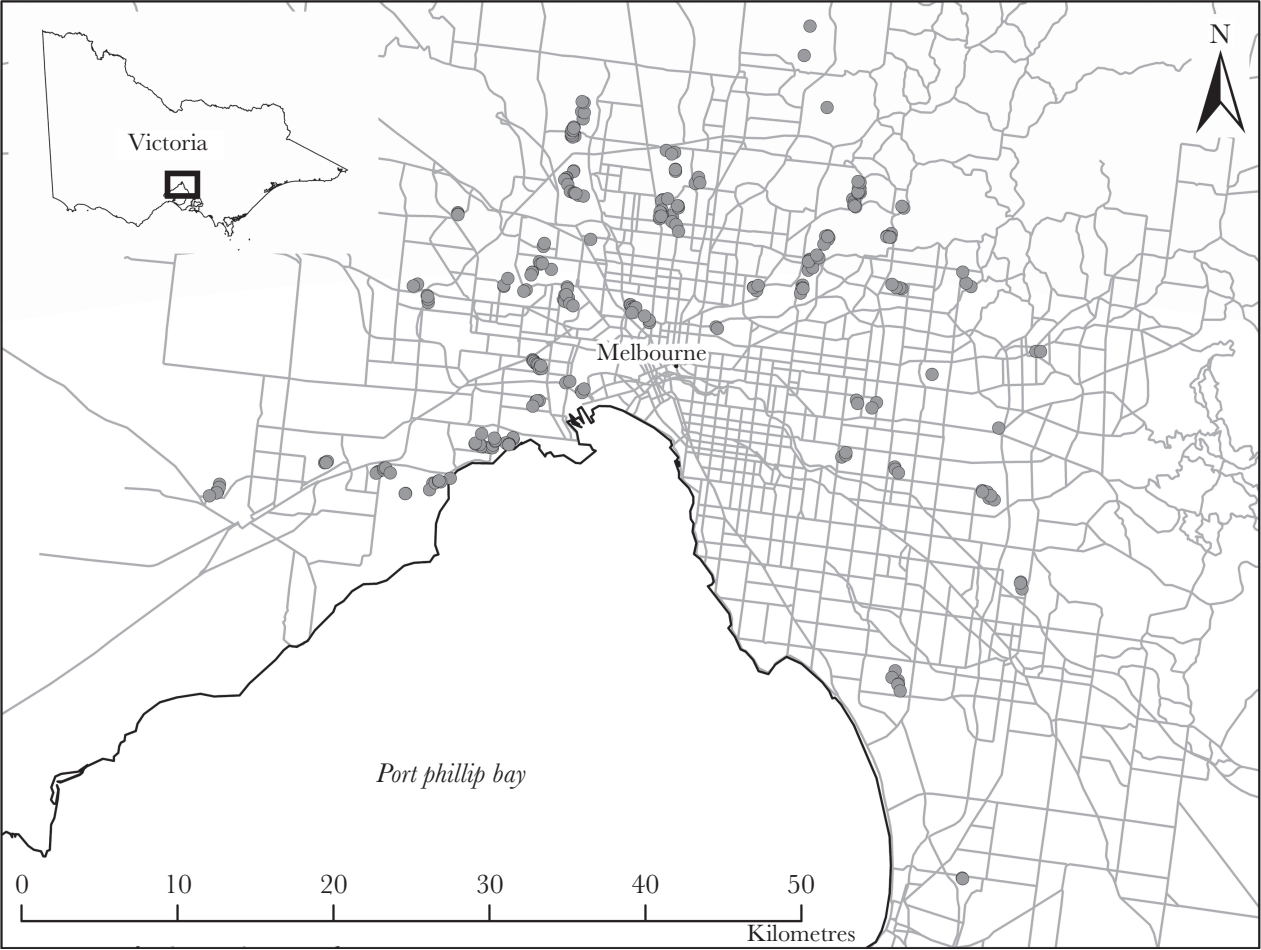
FID approaches (gray dots) conducted in the greater Melbourne area, Victoria, Australia. (Data source: VicMap Lite, Department of Environment, Land, Water, and Planning).

To index avian escape responses, we conducted “approaches” (which evoked escape) and enabled the quantification of escape initiation, distance fled, and connectivity of habitat. All approaches were conducted across 69 urban parks and reserves across metropolitan Melbourne (Victoria, Australia; Figure 1). To examine the influence of habitat connectivity on avian escape, we required species which used fragmented terrestrial habitats and for which connectivity could be indexed. We therefore targeted woodland birds (i.e., those that usually or often use trees; Supplementary Table S1) and avoided sampling strictly “open country” birds (after Garnett et al. 2015). Sites therefore contained a mosaic of remnant forest and revegetated habitat interspersed with open grassed areas (such as sports fields). Areas dominated by infrastructure that were lacking in suitable habitat for woodland birds were avoided. Fieldwork occurred during daylight hours from October 2021 until January 2022 and avoided periods of rain and high winds. Mean air temperature was 24.3 ± 0.2 °C (SE) (13.4–37.3 °C), and windspeed was 3.9 ± 0.2 km/h (0–19.3 km/h). Mean human activity across all sites, as measured by number of humans encountered per minute (i.e., within 50 m), was 0.48 ± 0.05, humans/min (0.00–1.78).

### Flight-initiation distance and pre-escape habitat connectivity

Flight-initiation distance (FID) measures were based on established protocols summarized by Blumstein and Cooper (2015) and Weston et al. (2012). Briefly, for each collection of an FID, a bird or single-species group of birds (“focal bird/s”) was selected according to a set of criteria. To qualify for sampling, focal birds were distant from other people and predators, not in close proximity to roads and water bodies, and on the ground or on perches (both natural and anthropogenic) of ≤10 m above ground, to increase the likelihood of an explicit escape response (*sensu*Cooper and Avalos 2010). Once an appropriate focal bird was identified, the distance between the approaching individual and the focal bird at the commencement of the approach was measured (i.e., starting distance, “StD”; Cooper and Blumstein 2015). One of us (M.R.) then commenced the approach by walking toward the focal bird/s at c. 1 ms^−1^, stopping when the focal bird/s began exhibiting escape behavior. Escape was determined when the bird interrupted its preceding activity to move away by walking, running, or flying. Distance between the bird’s initial position and the observer (i.e., FID) was then recorded. If the bird did not respond at the point where the observer was at the bird’s initial position, this was recorded as a “non-response” (6.2% of 499 approaches) and was excluded from analysis. In some cases (e.g., for birds partially obscured by vegetation, rendering distances from the rangefinder unreliable), we measured the horizontal distance to the bird’s perch and subsequently calculated the straight-line distance accounting for observer height, as do distances measured by a rangefinder (*sensu*Livezey and Blumstein 2016). We did not conduct multiple approaches on the same individual, and never sampled the same species within 50 m of previous sampling of that species, to reduce the risk of repeat sampling. Distances were measured using a Yukon Extend LRS-100 Laser Range Finder (accuracy ± 1 m).

To measure habitat connectivity in relation to each bird’s escape response, we calculated the density of perches available to focal birds at their initial position prior to the commencement of an approach. We use this metric because birds rarely flew to the closest available perch (distance to nearest refuge is used by others; e.g., Tätte et al. 2018), and the concept of “refuge” may not apply to animals escaping substantial distances through habitats rich in locations where escape can cease, meaning that “nearest refuge” may be irrelevant to escape (see below). Instead, perch density indexes available perches at the point of escape, across all directions available for escape. Alternative metrics (e.g., the proportion of cover within a given area surrounding a bird) rely on assumptions regarding the spatial scale at which connectivity is judged by birds, while perch density is based on perches within a bird’s line of sight.

Perches in this case were defined as any solid surface capable of supporting a bird, and both natural and human-made structures were considered to meet this definition, including trees, shrubs, fences, buildings, and powerlines. We adapted the point-centered quarter method (PCQ; Cottam and Curtis 1956) which enabled estimation of perch density (i.e., the density of shrubs, trees, or human infrastructure capable of offering perches). Hence, we measured the distance to the nearest available perch in each of four quadrants, demarcated by the four cardinal points, and derived the inverse of the mean of these values.

### Quantifying post-initiation elements of escape

Once a bird had commenced escape, it was tracked to its “refuge” (the location at which the bird stopped after its first escape movement) where possible (97.7% of 468 flight responses). Coordinates were taken by GPS at this point and at the bird’s initial position, except for birds that flew ≤5 m. For these birds, the initial position and refuge location were considered the same. The distance between the initial location and refuge location (i.e., “distance fled”) was also measured.

**Figure 2:**
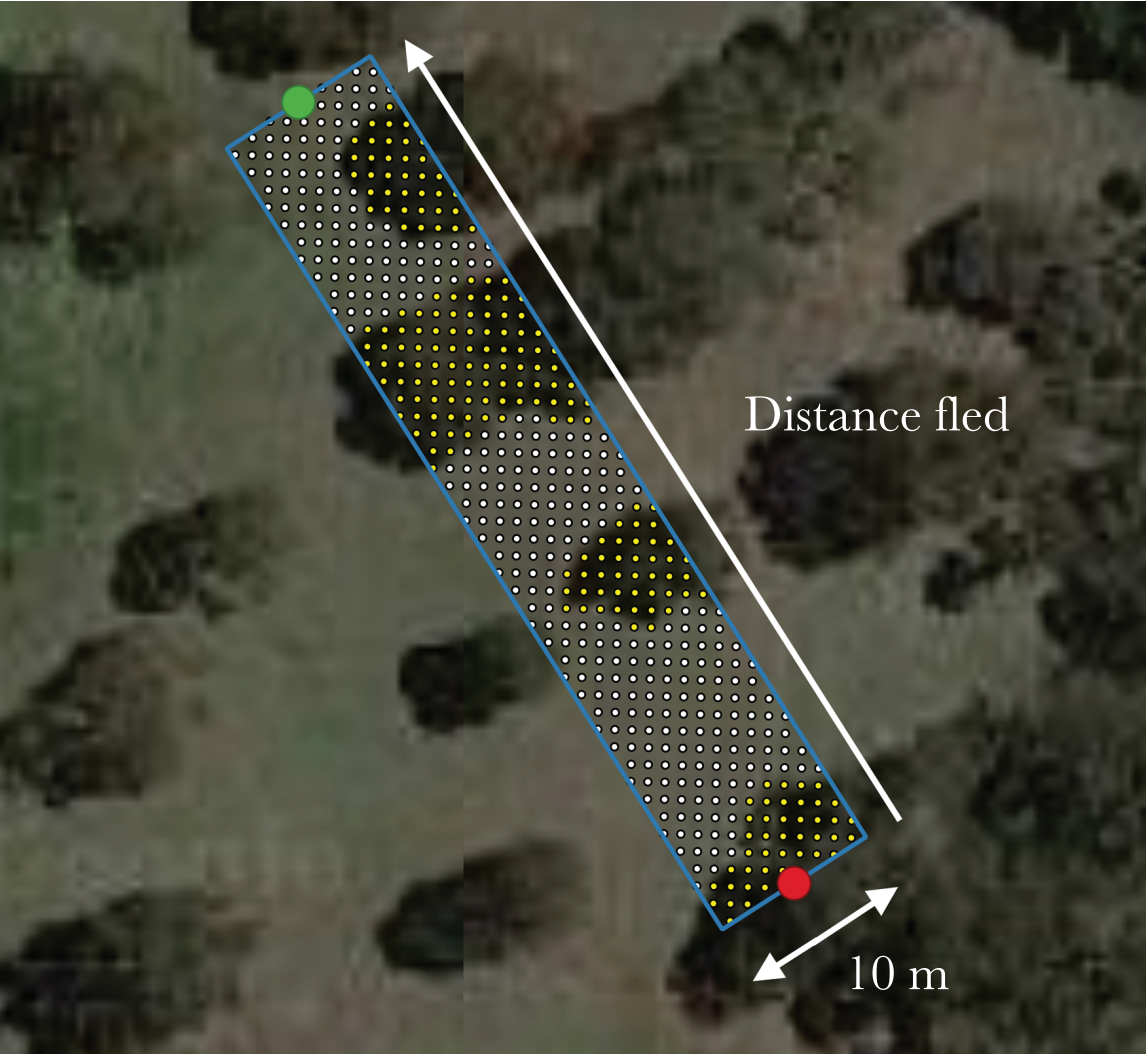
Example of how we quantified route connectivity during escape. This particular bird (a Gray Butcherbird, *Cracticus torquatus*) was not a part of this study and is used for illustrative purposes only. Red points indicate the bird’s initial position, green points indicate refuge location. Points (centers of 1 × 1 m grids) are spaced 1 m apart. White points represent grids not containing vegetation, yellow points represent grids containing vegetation. This encounter was given an escape route connectivity value of 0.43.

To determine whether habitat connectivity influenced the post-escape response of birds, we estimated connectivity along the escape route of each bird that flew *>*5 m. In this way, the relevant measure of connectivity, as defined by the focal bird’s escape route, was measured rather than a general measure of connectivity at the bird’s initial location (which contained areas within which connectivity was irrelevant to the escape). Measurements of “route connectivity”, as defined by the proportion of vegetation/perchable infrastructure coverage along the escape route of each bird (which moved *>* 5 m), were calculated in ArcMap version 10.7.1 (World Imagery source: Maxar) and QGIS (image resolution = 30 m; Gorelick et al. 2017). A 10 m wide rectangle was drawn between initial and refuge locations, running the length of the escape route (all escape flights occurred within this area; pers. obs.). This was used to define the route used during escape. Points were then overlaid in a 1 × 1 m grid over each escape route. We then counted the number of points within each escape route that were within some level of vegetation providing refuge perches and cover for fleeing birds (i.e., trees, shrubs, and infrastructure), reported as the proportion of all points within the escape route (Figure 2). Birds that moved ≤5 m from their initial position were given a route connectivity value of 1.0; that is, their route had 100% habitat connectivity.

Calculating the proportion of cover does not necessarily equate to habitat connectivity; rather it may be more reflective of the level of fragmentation (Tischendorf and Fahrig 2000). However, since we considered habitat use by woodland birds in the context of escape response, in which protective cover and refuge perches are integral aspects, and since many studies have found that structural characteristics and amount of habitat are better predictors of animal movement than patch matrix elements (Goodwin and Fahrig 2002; Jackson and Fahrig 2016), the measurements of perch density and route connectivity described above were deemed appropriate proxies for habitat connectivity in this study.

### Statistical analysis

We removed instances when birds did not respond to the approach (i.e., non-responses) and when the bird moved away by means other than flying (0.6% of 499 approaches). We also removed data for which the refuge location of the bird could not be determined in the field (2.3% of 468 flight responses). Species that subsequently had *>* 20 FIDs (*n* = 5) were considered for statistical analysis.

To test the “habitat connectivity hypothesis”, we built two generalized linear mixed models for each species pertaining to 1) escape response, and 2) post-initiation aspects of escape (i.e., escape trajectory and refuge). The first model tested whether the FID of woodland birds is influenced by the level of habitat connectivity at their initial position (“perch density”) and along their escape route (“route connectivity”). Starting distance (StD) was also included for its known influence on FID (Blumstein 2003). The park (location) in which the approach occurred was included as a random factor to account for similarities in human activities which might occur between parks and influence escape responses.

The second set of generalized linear mixed models considered the post-initiation elements of escape, and therefore tested for an effect of habitat connectivity and FID on the distance fled when flushed, as well as an interaction between these variables. “Perch density” was included as the measure of habitat connectivity at the point from which escape was initiated, and “route connectivity” was included as the measure of habitat connectivity during escape. Since FID is highly positively correlated with StD, including both as separate predictor variables in a single model would likely result in unacceptably high variance inflation factors. We therefore adjusted each FID value to account for StD (*sensu*Glover et al. 2015). Adjusted FID values (hereafter “FID_Adj._”) are the expected FID at the mean StD for each species, derived by adjusting each FID value according to the slope of a linear regression between FID against StD (“Slope_FID/StD_”). Specifically, FID_Adj._ is calculated for each species as follows: FID_Adj._ = FID_Observed_ + Slope_FID/StD_*(x̄StD – StD_Observed_).

Interaction terms between “perch density” and FID_Adj._, and between “route connectivity” and FID_Adj._ were included to test whether an effect of habitat connectivity pre- and post-initiation of escape is dependent on FID, regarding relation to the distance fled by the focal bird. Location was included as a random factor to account for locational differences that may influence escape response.

Statistical analysis was implemented in R (R Core Team 2020), using the “lme4” (Bates et al. 2015) and “MuMIn” (Bartoń 2022) packages. For each set of models, predictor variables were scaled and subsequently checked for issues of collinearity; however, no two predictor variables in any given model had correlations above 0.5, and so problems with collinearity did not exist. We specified Gaussian and/or Poisson distributions as determined through visual assessment of histograms with these subsequently validated through assessments of dispersion and residual plots (specified distributions are provided in Tables 1 and 2). Where overdispersion was present, a negative binomial distribution was used. Model validation further involved visual inspection of residual values plotted against fitted values as well as residuals plotted against each variable in the model. Cook’s distance values were used to assess the relative influence of individual data points on model outputs. Where overly influential points existed, these were removed, and models re-run to assess their leverage on qualitative findings. We considered effects as variables with a *P*-value of <0.05; or as trends if the *P*-value is < 0.10 but greater than 0.05. *P*-values 0.05–0.10 do not meet the usual threshold for a “statistical difference”, but instead suggest a “trend” toward an effect which might be realized with increased sampling.

**Table 1:** Results of species-specific generalized linear mixed models exploring the influence of habitat connectivity at both the point of escape (perch density) and post-escape (route connectivity) on the FID of five woodland bird species. One model was created for each species. RC is escape route connectivity; PD is perch density at the initial location. Statistically significant results are emboldened, trends are italicized

Model	Parameter	Estimate	SE	*z*-value	*P*-value	Lower CI	Upper CI
Noisy miner (negative binomial)	RC	−0.028	0.058	−0.481	0.631	−0.141	0.086
	PD	**0.127**	**0.060**	**2.122**	**0.034**	**0.010**	0.244*
Red Wattlebird (negative binomial)	RC	−0.128	0.066	−1.925	*0.054*	−1.428	1.172
	PD	−0.079	0.071	−1.112	0.266	−0.218	0.060
Common Myna (Poisson)	RC	−0.082	0.049	−1.674	*0.094*	−0.178	0.014
	PD	−0.098	0.058	−1.689	*0.091*	−0.212	0.016
Spotted Dove (Gaussian)	RC	−0.944	1.241	−0.761^^^	0.481	−3.376	1.488
	PD	−1.243	1.173	−1.060^^^	0.338	−3.542	1.056
New Holland Honeyeater (Poisson)	RC PD	−0.016 −0.020	0.108 0.116	−0.145 −0.169	0.853 0.892	−0.228 −0.247	0.196 0.208

^^^A *t*-value.

*A significant result.

**Table 2:** Results of generalized linear mixed models of influence of FID_Adj._ and habitat connectivity factors on distance fled for five woodland bird species. RC is escape route connectivity; PD is perch density at the initial location. Statistically significant results are emboldened, trends are italicized

Model	Parameter	Estimate	SE	*z*-value	*P*-value	Lower CI	Upper CI
Noisy miner (negative binomial)	FID_Adj._	0.067	0.096	0.690	0.490	−0.122	0.256
	RC	−**0.738**	**0.078**	−**9.455**	**<0.001**	**−0.891**	−0.585*
	PD	0.076	0.172	0.440	0.660	−0.262	0.413
	FID_Adj._*RC	−0.097	0.081	−1.195	0.232	−0.256	0.062
	FID_Adj._*PD	−0.137	0.259	−0.531	0.595	−0.645	0.370
Red Wattlebird (negative binomial)	FID_Adj._	0.088	0.108	0.813	0.416	−0.124	0.299
	RC	−**0.505**	**0.118**	**−4.270**	**<0.001**	**−0.737**	−0.273*
	PD	−0.093	0.130	−0.710	0.478	−0.348	0.163
	FID_Adj._*RC	0.063	0.116	0.541	0.588	−0.165	0.291
	FID_Adj._*PD	0.004	0.117	0.035	0.972	−0.226	0.234
Common Myna (Poisson)	FID_Adj._	**0.251**	**0.067**	**3.742**	**<0.001**	**0.119**	0.382*
	RC	**−0.508**	**0.077**	**−6.632**	**<0.001**	**−0.658**	−0.358*
	PD	−0.052	0.092	−0.563	0.573	−0.231	0.128
	FID_Adj._*RC	0.047	0.067	0.697	0.485	−0.085	0.179
	FID_Adj._*PD	−0.009	0.081	−0.109	0.913	−0.167	0.149
Spotted Dove (Poisson)	FID_Adj._	−0.079	0.086	−0.912	0.362	−0.248	0.090
	RC	**−0.404**	**0.103**	**−3.924**	**<0.001**	−0.606*	−0.202*
	PD	−0.194	0.119	−1.623	0.105	−0.427	0.039
	FID_Adj._*RC	−0.231	0.132	−1.745	*0.081*	−0.490	0.028
	FID_Adj._*PD	0.075	0.140	0.534	0.593	−0.199	0.349
New Holland Honeyeater	FID_Adj._	−0.243	0.264	−0.920^^^	0.400	−0.761	0.275
Gaussian; log transformed	**RC**	**−0.560**	**0.149**	**−3.764** ^^^	**0.013**	**−0.852**	−0.268*
	PD	−0.154	0.172	−0.896^^^	0.411	−0.491	0.183
	FID_Adj._*RC	0.251	0.686	0.365^^^	0.730	−1.094	1.595
	FID_Adj._*PD	−0.018	0.192	−0.094^^^	0.929	−0.395	0.359

^^^A *t*-value.

*A significant result.

## RESULTS

We collected 499 FIDs from 28 species across 69 locations (Supplementary Table S1). Five species met the minimum threshold of 20 samples for single-species analysis. These were: Noisy Miner *Manorina melanocephala* (*N* = 116), Red Wattlebird *Anthochaera carunculata* (*N* = 80), Common Myna *Acridotheres tristis* (*N* = 52), Spotted Dove *Spilopelia chinensis* (*N* = 24), and New Holland Honeyeater *Phylidonyris novaehollandiae* (*N* = 25).

### Effect of habitat connectivity on FID

Perch density at the point of escape had a positive influence on FID of Noisy Miners with birds taking flight at longer distances from the observer where more perches where available in their immediate vicinity (Table 1). Perch density trended towards a negative relationship with FID for Common Mynas, with longer FIDs observed where there was lower perch availability at the point of escape although confidence intervals overlapped zero (Table 1). There was no effect of perch density on FID of Red Wattlebird, Spotted Dove, or New Holland Honeyeater (Table 1). Route connectivity trended toward a negative relationship with FID of Red Wattlebirds and Common Mynas with lower connectivity along the bird’s escape route associated with longer FIDs although confidence intervals overlapped zero (Table 1). There was no effect of route connectivity on FID of Noisy Miner, Spotted Dove, or New Holland Honeyeater. StD was positively correlated with FID of all species (Supplementary Table S2 and Figure S1).

### Effect of habitat connectivity on distance fled

**Figure 3:**
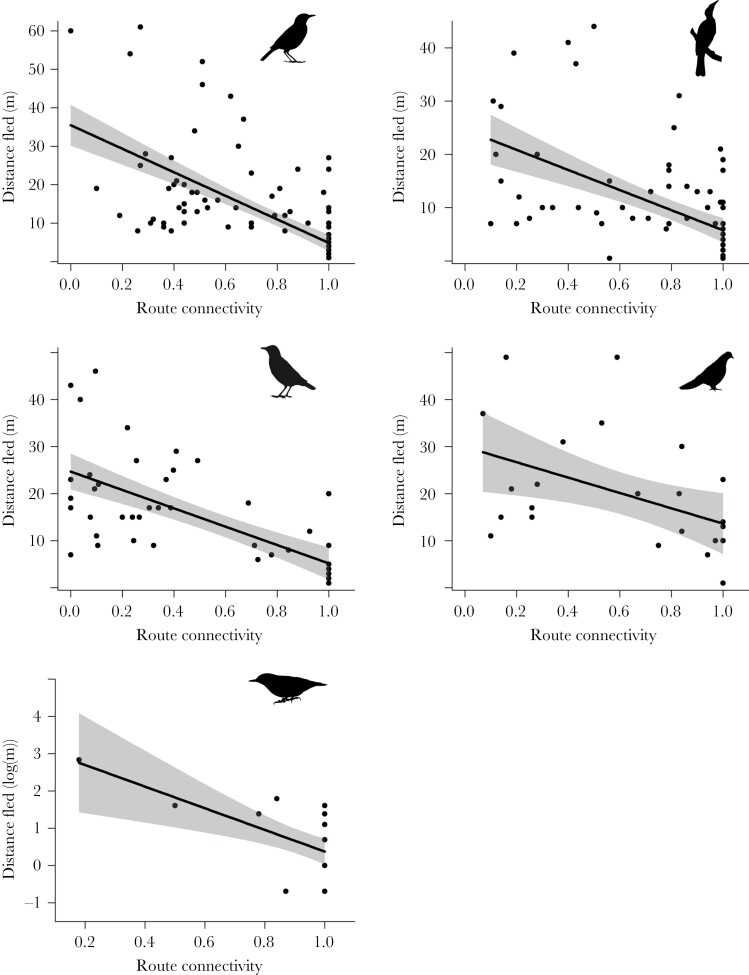
Effect of route connectivity on distance fled by five focal species. Top left: Noisy Miner; top right: Red Wattlebird; middle left: Common Myna; middle right: Spotted Dove; bottom left: New Holland Honeyeater.

Distance fled was strongly influenced by habitat connectivity along the escape route of fleeing birds, with lower escape route connectivity associated with longer distances fled for all species (Table 2; Figure 3). There was a positive effect of FID_Adj._ on distance fled in Common Myna with longer FID_ Adj_. resulting in longer distances fled (Table 2). There was no effect of FID_Adj._ on distance flown in Noisy Miner, Red Wattlebird, Spotted Dove, or New Holland Honeyeater. Perch density had no influence on distance fled by any of the species modeled (Table 2). The interactive effects of FID_Adj._ and route connectivity on distance fled trended towards a negative relationship for Spotted Dove (Table 2), with the combined effects of shorter FID and less habitat connectivity along the escape route resulting in longer distances fled, although confidence intervals overlapped zero. This was not evident in Noisy Miner, Red Wattlebird, Common Myna, or New Holland Honeyeater (Table 2). There was no effect of the interaction between FID_Adj._ and perch density on distance fled by any species (Table 2).

## DISCUSSION

We demonstrate that habitat connectivity can influence escape among urban woodland birds; namely that reduced connectivity exacts a higher cost of escape in terms of longer distances fled. This effect has been demonstrated in various lizards (Martin and Lopez 1995; Schulte et al. 2004; Maia-Carneiro et al. 2021), but to the best of our knowledge this is the first study that identifies this effect in flying birds. There was little support for an effect of habitat connectivity on FID as predicted by the habitat connectivity hypothesis other than the positive effect of perch density on FID for Noisy Miners, but this effect was not evident in the other species studied and was not in the predicted direction.

### Habitat connectivity influences distance fled

The nearest available perch was often not the one chosen as refuge by our study birds, instead most undertook longer escape flights. We found no effect of perch density on FID (but see Tätte et al. 2018, who report a positive relationship between distance to nearest perch and distance fled in 17 bird species), but the negative influence of escape route connectivity on distance fled was evident in all five species we studied. This suggests that lack of habitat connectivity imposes a higher cost of escape on woodland birds as predicted by the habitat connectivity hypothesis. The most obvious explanation for this result is that refugia are positioned at greater distances when habitat connectivity is low. The various habitat components used as refuge by the woodland birds studied here indicate that these species may not have strict requirements for selecting refugia (birds used trees, shrubs, infrastructure, and even the ground). Flexibility of refuge use and location may make it more difficult for a pursuing predator (*sensu*Martin and Lopez 1995). Indeed, the concept of refuge may not apply for woodland birds. Species which unambiguously use refuges seek constructed or naturally occurring shelter from predators in the form of burrows or cover, or transition between habitats such as climbing up trees or moving into water from land (Dill and Houtman 1987; Bonenfant and Kramer 1996; Hemmi 2005; Lagos et al. 2009; Guay et al. 2013; Dear et al. 2015).

Rather than selecting specific refuge locations or features, fleeing birds may be attempting to place a certain amount of protective cover between themselves and the approaching threat, thereby requiring greater distances flown in areas of low habitat connectivity. A related possibility is that they establish a temporal margin of safety, defined as the expected time between the fleeing animal and pursuing predator arriving at the refuge location (Blumstein and Cooper 2015), by placing more cover/obstacles between themselves and a predator, given that structure can impede predator pursuit (Wheatley et al. 2020). As such, rather than representing a refuge, the post-escape stopping point may merely represent the location at which a successful escape concludes.

In line with the results of Tätte et al. (2018), one but not our other study species exhibited a relationship between distance fled and FID. There was a positive effect of FID_Adj._ on distance fled only in Common Myna, with longer FID_Adj._ being associated with greater distances fled. It may be that heavier birds, realizing greater costs of escape, may exhibit positive relationships between FID and distance fled (Tätte et al. 2018), and we note that Common Myna was among the heaviest species we studied (Higgins and Davies 1996; Higgins et al. 2001; Higgins et al. 2006). For Common Myna, some individuals “flee early and flee far”, while others do not, perhaps suggesting the presence of behavioral syndromes, or a genetically mediated wariness (see van Dongen et al. 2015). Future research could consider a broader taxonomic scope and consider phylogenetic analyses to consider these relationships more robustly.

### Habitat connectivity does not influence FID for most species tested

FID was not unambiguously related to habitat connectivity in this study, although the predicted relationship was evident for one of the five species (Noisy Miner), and then only for perch density. Longer “distance from nearest refuge” may result in increased perceived predation risk, thereby increasing FID (Tätte et al. 2018) which would manifest as a negative correlation between FID and distance from nearest refuge. We measured perch density as a measure of connectivity at the site where escape commenced rather than distance to nearest perch (see Methods; distance to nearest perch was not highly correlated with our perch density measure; *r* = −0.29). Nevertheless, we also predicted negative association between FID and perch density. The only statistically significant association of FID and perch density (Noisy Miner) was in the opposite direction to that predicted; althougha trend in the expected direction existed for Red Wattlebird and Common Myna. For Common Myna, there was a trend towards a negative association between FID and route connectivity. While this requires verification from further study, it may suggest escape routes are known before escape commences in this species. The effect of nearest refuge on FID is clearly not universal among birds (Tätte et al. 2018), and nor evidently is the effect of perch density (or route connectivity, if at all). The species sampled here usually flew beyond the nearest perch during escape, suggesting that measures of refuge proximity at the bird’s initial location may not encompass the spatial availability of actual refuges. The positive relationship for perch density and FID in Noisy Miner defies unambiguous interpretation.

In general accordance with our results, Tätte et al. (2018) recorded no effect of vegetative cover within 30 m of a bird, or on the distance to the nearest refuge on FID of 17 bird species (concepts embedded within our measure “perch density”). One possible explanation for the lack of effect for the four species other than the Noisy Miner could relate to the failure of the habitat connectivity hypothesis to acknowledge the gradient of predator refuge selection which is evident across many animals. Animals range from refuge specialists (species that have a fixed place of refuge) to refuge generalists (species that do not have a fixed place of refuge). The abundance of refuge options which is apparent for our study species may mean that escape trajectories are not pre-planned or pre-experienced, potentially explaining the lack of relationship between FID and fine-scale habitat factors for most of the species studied, because the cost of escape would not then be known until escape commences. The fact that an association between FID and perch density was borne out in one of five species tested, suggests that comparative analyses of a larger taxonomic breadth may be useful in understanding what factors underly such interspecific variation.

### Implications

We demonstrate an additional cost of living in poorly connected habitats. The mediation of the efficiency of effective predator escape (i.e., distance fled) by habitat connectivity may be a mechanism which contributes to some of the larger scale patterns whereby depauperate avian diversity persists in poorly connected, fragmented contexts (Shanahan et al. 2011). Clearly, further study and a broader taxonomic sample is required to examine the generality of these findings. The configuration of habitat in urban parks influences the cost of escape from predators for at least some wildlife living there, and land-use planners might consider wildlife “escape routes” as part of other planned revegetation works. Well-planned revegetation works are likely to enhance predator populations in time, so such escape routes may facilitate the usage of parks by some wildlife.

## Supplementary Material

arac127_suppl_Supplementary_Material

## Notes

This research was conducted on the traditional lands of the Bunurong Boon Wurrung and Wurundjeri Woi Wurrung people of the Kulin nation. This research was approved by Deakin University Animal Ethics Committee (B08-2021) and the Department of Environment, Land, Water and Planning (10010123). Editorial work occurred at BEACH (Venus Bay).

Data Availability: Analyses reported in this article can be reproduced using the data provided by Weston et al. (2022).
