# Supplementary material for: The habitat connectivity hypothesis of escape in urban woodland birds
Source: Behav Ecol. 2023 Feb 10;34(2):297–305. doi: 10.1093/beheco/arac127 (PMC10047614; doi:10.1093/beheco/arac127)
Supplement: arac127_suppl_Supplementary_Material [file arac127_suppl_supplementary_material.docx]

**Supplementary Material**

**Table S1**. Summary statistics of total FID approaches. SE cannot be calculated for n = 1 sample sizes. Bold indicates species that were further analysed (focal species). Route connectivity values only calculated for five focal species.

| Species | Number of approaches | Number of locations | | | x̄ StD (m) | x̄ FID (m) | | SE | | Min FID (m) | | | Max FID (m) | | Number of 'no responses' | | x̄ distance fled (m) | | x̄ perch density | | x̄ route connectivity | |  |
| --- | --- | --- | --- | --- | --- | --- | --- | --- | --- | --- | --- | --- | --- | --- | --- | --- | --- | --- | --- | --- | --- | --- | --- |
| **Noisy Miner** (*Manorina melanocephala)* | **122** | **42** | | | **20** | **6** | | **0.428** | | **0** | | | **28** | | **5** | | **11** | | **0.059** | | **0.799** | |  |
| **Red Wattlebird** (*Anthochaera carunculata*) | **84** | **36** | | | **18** | **7** | | **0.523** | | **0** | | | **20** | | **0** | | **11** | | **0.061** | | **0.788** | |  |
| **Common Myna** (*Acridotheres tristis*) | **53** | **30** | | | **31** | **16** | | **1.034** | | **4** | | | **43** | | **0** | | **15** | | **0.021** | | **0.543** | |  |
| Rainbow Lorikeet (*Trichoglossus moluccanus*) | 35 | 25 | | | 16 | 3 | | 0.491 | | 0 | | | 9 | | 16 | | 6 | | 0.033 | | — | |  |
| **Spotted Dove** (*Spilopelia chinensis*) | **27** | **19** | | | **25** | **13** | | **1.135** | | **2** | | | **24** | | **0** | | **20** | | **0.093** | | **0.632** | |  |
| **New Holland Honeyeater** (*Phylidonyris novaehollandiae*) | 26 | 16 | | | 11 | 4 | | 0.475 | | 0 | | | 9 | | 1 | | 4 | | 0.202 | | — | |  |
| White-plumed Honeyeater (*Lichenostomus penicillatus*) | 21 | 15 | | | 14 | 7 | | 1.096 | | 0 | | | 20 | | 1 | | 13 | | 0.096 | | — | |  |
| Grey Butcherbird (*Cracticus torquatus*) | 20 | 15 | | | 18 | 9 | | 1.643 | | 0 | | | 28 | | 2 | | 22 | | 0.119 | | — | |  |
| Eastern Rosella (*Platycercus eximius*) | 18 | 14 | | | 28 | 15 | | 2.096 | | 1 | | | 33 | | 0 | | 28 | | 0.088 | | — | |  |
| Willie Wagtail (*Rhipidura leucophrys*) | 18 | 12 | | | 22 | 8 | | 0.865 | | 2 | | | 16 | | 0 | | 11 | | 0.084 | | — | |  |
| Little Raven (*Corvus mellori*) | 14 | 12 | | | 30 | 11 | | 1.966 | | 3 | | | 27 | | 1 | | 20 | | 0.008 | | — | |  |
| Little Wattlebird (*Anthochaera chrysoptera*) | 11 | 9 | | | 15 | 6 | | 1.224 | | 0 | | | 16 | | 0 | | 6 | | 0.487 | | — | |  |
| House Sparrow (*Passer domesticus*) | 9 | 7 | | | 20 | 11 | | 2.041 | | 4 | | | 20 | | 0 | | 17 | | 0.058 | | — | |  |
| Common Blackbird (*Turdus merula*) | 7 | 5 | | | 18 | 8 | | 1.746 | | 4 | | | 17 | | 0 | | 10 | | 0.021 | | — | |  |
| Galah (*Eolophus roseicapilla*) | 5 | 4 | | | 24 | 9 | | 2.059 | | 3 | | | 14 | | 0 | | 17 | | 0.054 | | — | |  |
| Common Starling (*Sturnus vulgaris*) | 4 | 4 | | | 27 | 16 | | 4.308 | | 5 | | | 26 | | 0 | | 16 | | 0.025 | | — | |  |
| Laughing Kookaburra (*Dacelo novaeguineae*) | 4 | 4 | | | 23 | 14 | | 3.014 | | 8 | | | 20 | | 1 | | 21 | | 0.005 | | — | |  |
| Musk Lorikeet (*Glossopsitta concinna*) | 4 | 4 | | | 11 | 6 | | — | | 6 | | | 6 | | 3 | | 14 | | 0.006 | | — | |  |
| Red-rumped Parrot (*Psephotus haematonotus*) | 4 | 4 | | | 19 | 7 | | 0.577 | | 6 | | | 8 | | 1 | | 11 | | 0.027 | | — | |  |
| Pied Currawong (*Strepera graculina*) | | | 3 | 2 | | | 14 | | 3 | | 2.028 | 0 | | 7 | | 0 | | 22 | | 0.174 | | — | |
| Sulphur-crested Cockatoo (*Cacatua galerita*) | | | 3 | 3 | | | 42 | | 14 | | 8.021 | 5 | | 30 | | 0 | | 52 | | 0.005 | | — | |
| Black-faced Cuckoo-shrike (*Coracina novaehollandiae*) | | | 1 | 1 | | | 12 | | 7 | | — | 7 | | 7 | | 0 | | 34 | | 0.009 | | — | |
| Common Bronzewing (*Phaps chalcoptera*) | | | 1 | 1 | | | 15 | | 4 | | — | 4 | | 4 | | 0 | | 18 | | 0.071 | | — | |
| Crested Pigeon (*Ocyphaps lophotes*) | | | 1 | 1 | | | 7 | | 7 | | — | 7 | | 7 | | 0 | | 17 | | 0.007 | | — | |
| Dusky Woodswallow (*Artamus cyanopterus*) | | | 1 | 1 | | | 13 | | 9 | | — | 9 | | 9 | | 0 | | 48 | | 0.010 | | — | |
| European Greenfinch (*Chloris chloris*) | | | 1 | 1 | | | 40 | | 28 | | — | 28 | | 28 | | 0 | | 15 | | 0.010 | | — | |
| Sacred Kingfisher (*Todiramphus sanctus*) | | | 1 | 1 | | | 41 | | 13 | | — | 13 | | 13 | | 0 | | 16 | | 0.005 | | — | |
| White-faced Heron (*Egretta novaehollandiae*) | | | 1 | 1 | | | 42 | | 9 | | — | 9 | | 9 | | 0 | | 11 | | 0.017 | | — | |

**Table S2**. Results of species-specific generalised linear mixed models exploring the influence of starting distance (StD) on the FID of five woodland bird species.

| Model | Estimate | SE | z-value | Lower CI | Upper CI |
| --- | --- | --- | --- | --- | --- |
| Noisy Miner  (negative binomial) | 0.374 | 0.057 | 6.527 | 0.262* | 0.486 |
| Red Wattlebird  (negative binomial) | 0.286 | 0.066 | 4.303 | 0.157* | 0.415 |
| Common Myna  (Poisson) | 0.212 | 0.043 | 4.970 | 0.128* | 0.296 |
| Spotted Dove  (Gaussian) | 2.465 | 1.142 | 11.788^ | 0.227* | 4.703 |
| New Holland Honeyeater  (Poisson) | 0.235 | 0.089 | 2.635 | 0.060* | 0.411 |

^Indicates a t-value

* Indicates a statistically significant result

**
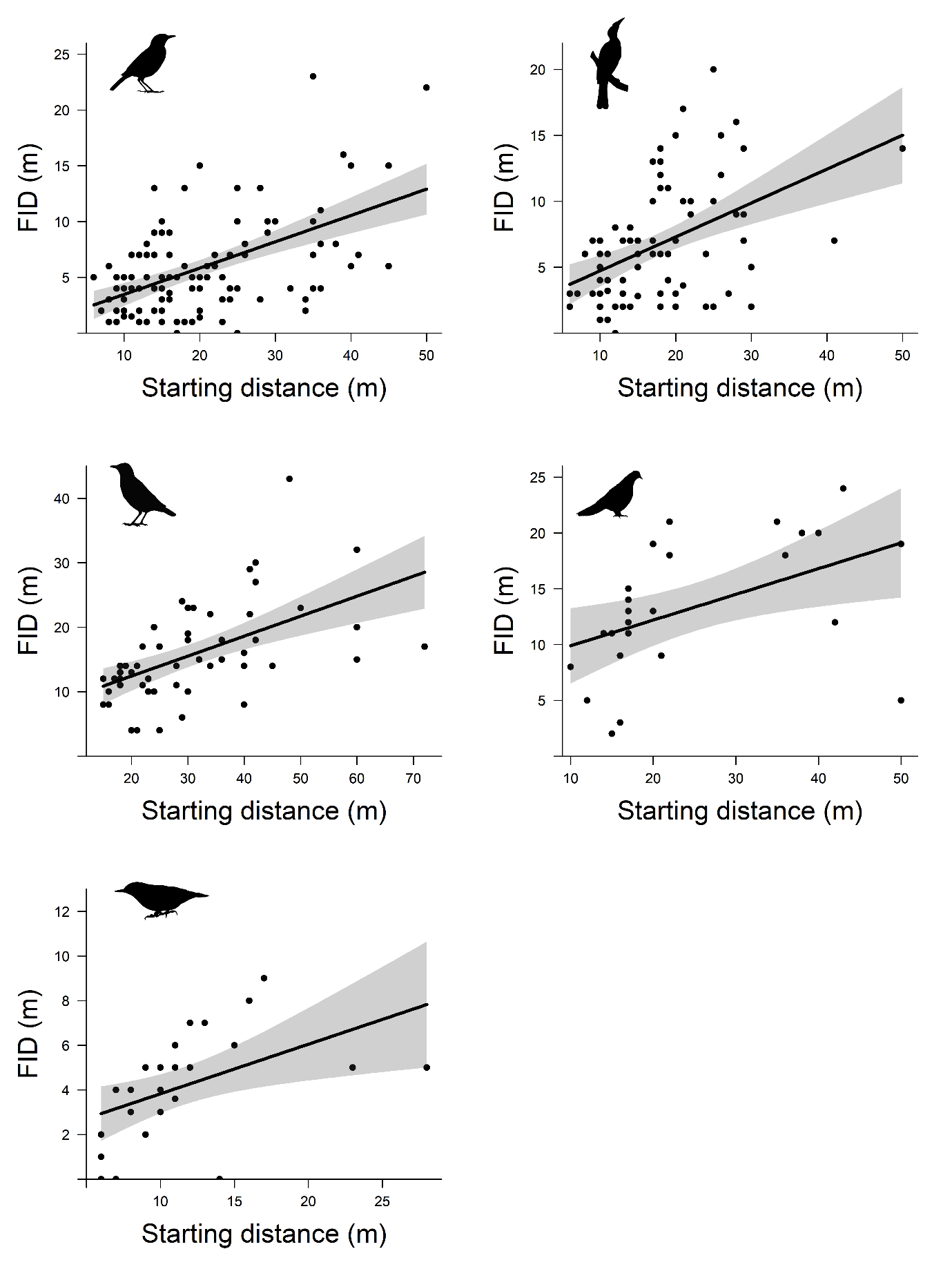
**

**Figure S1**. Effect of starting distance on FID by five focal species. Top left: Noisy Miner; top right: Red Wattlebird; middle left: Common Myna; middle right: Spotted Dove; bottom left: New Holland Honeyeater.
